# An Experimental and Analytical Study on a Damage Constitutive Model of Engineered Cementitious Composites under Uniaxial Tension

**DOI:** 10.3390/ma15176063

**Published:** 2022-09-01

**Authors:** Dapeng Zhao, Changjun Wang, Ke Li, Pengbo Zhang, Lianyou Cong, Dazhi Chen

**Affiliations:** 1 Department of Civil Engineering, Zhengzhou University, Zhengzhou 450001, China; 2 Henan Urban Planning Institute and Corporation, Zhengzhou 450044, China

**Keywords:** engineered cementitious composites, damage constitutive model, stress–strain relationship, tensile performance, monotonic uniaxial tensile test

## Abstract

Engineered cementitious composites (ECC) exhibit ultra-high ductility and post-cracking resistance, which makes it an attractive material in civil engineering. First, a monotonic uniaxial tensile test was performed, considering the effects of polyvinyl alcohol (PVA) fiber volume content and water-binder ratio. Then, the effects of the above variables on the tensile characteristics including the tensile stress–strain relationship, deformation capacity, and fracture energy were investigated based on test results; and when the water-binder ratio is 0.28 and the fiber volume content is 2%, the deformation performance of ECC is improved most significantly. Next, combined with damage mechanics theory, the damage evolution mechanism of ECC in monotonic uniaxial tension was revealed, based on which the damage factor and damage evolution equation of ECC were developed and the expressions of model parameters were proposed. Moreover, the comparison between the proposed model and test results demonstrated the accuracy of the proposed model. Finally, to further verify the feasibility of the proposed model, a finite element (FE) simulation analysis of the tensile performance of high-strength stainless steel wire rope (HSSWR) reinforced ECC by adopting the proposed model was compared with test results and the simulation analysis results by using anther existing model, the “trilinear model of ECC”. The comparison shows that the proposed model in this paper can predict more accurately.

## 1. Introduction

Engineered cementitious composites (ECC) is a new type of fiber reinforced cementitious composite material proposed in the 1990s and continuously developed in recent decades [1,2,3], which mainly consists of cement, sand, fly ash, silica fume, fibers, and water. The fibers used in ECC, which play a crucial role in improving the material performance [4], commonly include polyvinyl alcohol (PVA) [5,6,7], polypropylene (PE) [8,9,10], PP [11,12,13], and hybrid fibers [14,15,16]. Besides, studies have shown that adding new additives, such as eggshell powder [17] or alkali-activated products composed of industrial waste materials [18], can improve the mechanical properties of cementitious materials and increase environmental protection, which may also be used for improving ECC performance. Due to the presence of fibers, ECC exhibits ultra-high toughness, ductility, and crack-control capacity, which make ECC widely applied to constructing ductile structures and strengthening existing structures [19,20].

Many researchers have focused on the constitutive model of ECC in tension in order to give an accurate analytical calculation or numerical analysis of mechanical properties of structures constructed using ECC [21,22,23,24,25,26]. A trilinear model for charactering the stress–strain relationship of the ideal strain-hardening material, such as ECC, was first proposed by Li [21], based on which Kanda [22] proposed a simplified bilinear model of the tensile stress–strain curve of ECC. However, these two models did not consider the strain-softening stage of ECC. Then, a simplified trilinear model including a pre-cracking linear elastic section, a strain-hardening section and a strain-softening section has been widely adopted for more accurate FE numerical simulations of the whole-process mechanical behavior of ECC in tension [23,24,25,26,27,28]. However, this model ignores the nonlinear characteristics of the stress–strain relationship and cannot describe the damage evolution of ECC.

Damage modelling is a new approach to describe the mechanical behavior of materials by using damage mechanics theories [29,30,31], which attributes the fracture failure of material to the development of microscopic defects in materials in the whole loading process by employing damage factors to express the damage degree. Therefore, in recent years, damage models were applied to fiber-reinforced cementitious composites, such as ECC and fiber reinforced concrete [32,33,34,35,36]. The commonly used methods for defining damage factors include the stiffness method [32,33,34], the stress method [27], and the energy method [35,36]. However, these methods of defining damage factor do not clearly reveal the damage mechanism of ECC during the whole tension process, nor do they clearly reveal the role played by fibers in the damage process of ECC.

Therefore, in this paper, the damage constitutive model of ECC under uniaxial tension was studied experimentally and analytically. The main contents and purposes of this study include: (1) to evaluate the effects of PVA fiber volume content and water-binder ratio on the tensile properties of ECC under uniaxial tensile test; (2) to investigate the damage evolution law of ECC under uniaxial tensile; (3) to develop the damage constitutive model of ECC under uniaxial tension based on the test results; (4) to validate the acceptability and accuracy of the proposed model through FE analysis of high-strength stainless steel wire rope (HSSWR) reinforced ECC under uniaxial tension by adopting this model and existing trilinear model.

## 2. Test Procedure

### 2.1. Test Material and Specimen Production

The component materials of ECC used in this paper are ordinary silicate cement, fly ash, micro-silica fume, silica sand, PVA fiber, superplasticizer, and water. The mix proportions of the test specimens are shown in Table 1. The performance indicators of PVA used in this test are provided by Japan Kuraray Co., Ltd. (Tokyo, Japan), as shown in Table 2.

**Figure 1:**
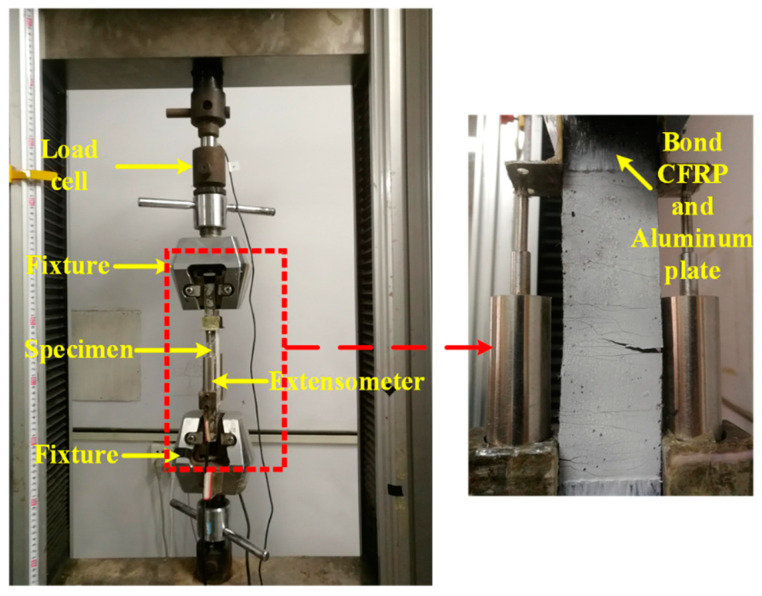
Test setup.

In this test, five groups of specimens with thin plate dimensions of 280 mm (length) × 40 mm (width) × 13 mm (thickness) were used, and each group consisted of 5 identical specimens. The volume content of PVA fibers (*v*) and the water-binder ratio (*r*) were considered, as listed in Table 3. It is worth noting that, according to the ready-mix test, when the water-binder ratio is less than 0.24, the matrix is too dry to mix, and when the water-binder ratio is greater than 0.3, the matrix is too dilute to be used normally, so the water-binder ratio of 0.24–0.28 was selected. Considering performance improvement efficiency [2,37,38,39,40,41] and economy [42], the fiber volume content was selected as 1%, 1.5% and 2%. The production process of the test specimen can be briefly summarized as follows: first, weigh the materials according to the mix proportion in Table 1, then mix the dry materials (cement, sand, fly ash, micro-silica fume) for 3 min; next, add water and superplasticizer and mix for 3 min; finally, add the PVA fibers evenly and continue to mix for 3 min. After 28-day curing in saturated lime water, the specimens were taken out and dried. Then, the two ends of the specimens to be clamped by the loading device were reinforced firstly by being externally bonded by carbon fiber reinforced polymer (CFRP) sheet, as shown in Figure 1. Specifically, firstly the two ends of the specimens were polished with sandpaper and cleaned with alcohol. Then, the two ends of the specimen to be clamped were externally bonded by one layer of CFRP sheets using epoxy resin adhesive. Finally, the 0.8-mm thick aluminum plates were bonded to the surface of the CFRP sheets using epoxy resin adhesive. A seven-day curing of the epoxy resin adhesive to make the adhesive reach a certain strength before the ECC tensile test was carried out. The tested tensile strength and elastic modulus of the used CFRP sheet with nominal thickness of 0.111 mm were 4150 MPa and 225 GPa, respectively. As given by the manufacturer, the tensile strength and elastic modulus of the used epoxy resin adhesive were 47.2 MPa and 2860 MPa, respectively.

### 2.2. Test Loading and Measurement

The test setup and loading device is shown in Figure 1. The test was carried out on a 10-ton electro-hydraulic servo material testing machine produced by Jinan Sans Dynamic Testing Technology Co., Ltd. (Jinan, China). The displacement-controlled monotonic loading method with a speed of 0.2 mm/min was adopted. The average temperature and humidity of laboratory are 298.15 K and 95%, respectively. Strain gage was placed on the center of the specimen surface, which was used mainly to measure the strain change of the specimen before cracking. The extensometer was placed at the middle region of the specimen with a measuring range of 120 mm, which was mainly used to obtain the specimen strain after cracking by dividing the measured displacement by the measuring range.

## 3. Test Results and Discussion

### 3.1. Analysis of Tensile Mechanical Properties Indicators

**Figure 2:**
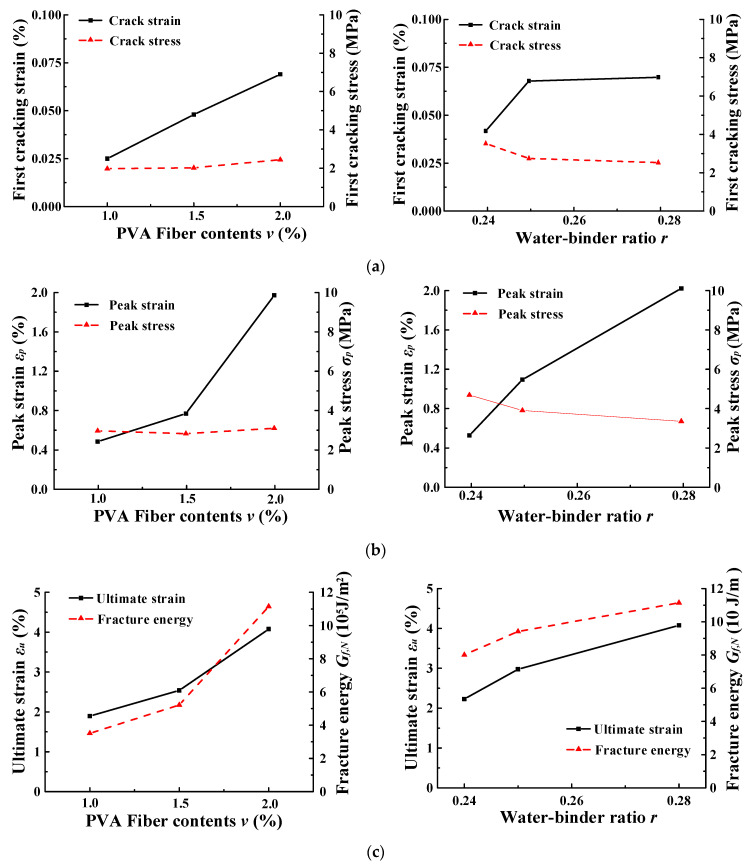
ECC tensile properties: (**a**) First cracking strain and stress; (**b**) Peak strain and stress; (**c**) Ultimate strain and fracture energy.

The tensile mechanical property indicators including first-cracking strain (*ε_k_*), first-cracking stress (*σ_k_*), peak strain (*ε_p_*), peak stress (*σ_p_*), ultimate strain (*ε_u_*), and fracture energy (*G_f_*) for each group of specimens are shown in Table 3. The relationship between the tensile mechanical property indicators and the volume content of PVA fibers (*v*) or the water-binder ratio (*r*) are shown in Figure 2. The test results were summarized in Table 3, and two specimens in Group V3R1 were excluded due to the large deviation of the test specimen results.

#### 3.1.1. First-Cracking Strain and Cracking Stress

The first cracking point was detected by the first drop point of the stress–strain curve of ECC under uniaxial tension. As presented in Table 3 and Figure 2a, when the volume content of PVA fibers was increased from 1.0% to 1.5% and 2% respectively, the crack stress increased by 2.2% and 21.1%, respectively, and the first crack strain increased by 92.0% and 43.8%, respectively. This indicates that increasing the volume content of PVA fibers in the range of 1.0–2% can effectively improve the crack resistance of ECC. When the water-binder ratio of ECC was increased from 0.24 to 0.25 and 0.28, respectively, the crack stress decreased by 22.5% and 8.2%, respectively, but the first crack strain increased by 63.4% and 3%, respectively. This indicates that increasing the water-binder ratio would decrease the first-cracking stress, but it can increase the first-cracking strain. This may be because the increase of the water in the matrix would make the interfacial bond strength between the matrix and fibers decrease, which results in the increase of the interfacial relative slip between the fibers and matrix [37].

#### 3.1.2. Peak Strain and Peak Stress

According to Table 3 and Figure 2b, when the volume content of PVA fibers was increased from 1.0% to 1.5% and 2% respectively, the peak stress of ECC increased by −4.7% and 9.3% respectively, and the peak strain increased by 55.8% and 151.3% respectively. When the water-binder ratio of ECC was increased from 0.24 to 0.25 and 0.28 respectively, the peak stresses decreased by 17.2% and14.6% respectively, but the peak strains increased by 112.9% and 86.7% respectively. This indicates that the peak stress of ECC was mainly determined by the water-binder ratio of the matrix, and the increase in fiber content would not significantly increase the peak stress. However, the increase in either PVA fiber content or water-binder ratio could significantly increase the peak strain. Similar phenomenon also appeared in Li’s investigation [38]. This may be because the increase of the fiber content in the matrix can make the cracks disperse to more cross sections, and as result the relative slip between fiber and matrix increased [1,2,3]. In addition, as discussed above, the increase of the water-binder ratio would make the interfacial frictional force between the matrix and fibers decrease, which results in an increase of the interfacial relative slip.

#### 3.1.3. Ultimate Strain

The ultimate strain is the strain at which the ECC specimens rupture, which reflects the maximum deformation capacity of ECC. The ultimate strain of each group of specimens is shown in Table 3. The relationship between the ultimate strain of ECC and the PVA fiber volume content or the water-binder ratio is shown in Figure 2c. According to Table 3 and Figure 2c, the ultimate strain of ECC increases by 34.0% and 60.6% when the volume content of PVA fiber increased from 1.0% to 1.5% and 2%, respectively. The ultimate strain of ECC increased by 33.4% and 37.0%, when the water-binder ratio of ECC increases from 0.24 to 0.25 and 0.28, respectively. This indicates that increasing the content of PVA fibers or the water-binder ratio could enhance the deformation capacity of ECC, and the enhancing effect of increasing the content of PVA fibers is more significant.

#### 3.1.4. Fracture Energy under Tension

Tensile fracture energy is defined as the energy required per unit area of crack extension, and it is a key indicator to describe the crack-control and energy dissipation capabilities of a material [39,40,41]. According to the calculation method of fracture energy in the literature [41], the total tensile fracture energy (*G_f,N_*) of a specimen can be expressed as:

(1) $$G_{f,N}=G_{f,A,N}+G_{f,B,N}$$

where

(2) $$G_{f,A,N}=\left[\int_{0}^{\varepsilon_{p}}\sigma \left(\varepsilon \right)d\varepsilon -\frac{1}{2}\frac{\sigma_{p}{}^{2}}{E}\right]L_{g}$$

(3) $$G_{f,B,N}=\int_{\varepsilon_{res}L_{g}}^{\varepsilon_{p}L_{g}}\sigma \left(\delta \right)d\delta$$

(4) $$\varepsilon_{res}=\varepsilon_{p}-\frac{\sigma_{p}}{E_{0}}$$

where *L_g_* is the gauge length and *E*_0_ is initial tensile modulus.

The calculated result of tensile fracture energy of ECC for each specimen was calculated and given in Table 3. The effects of PVA fiber volume content and water-binder ratio on the tensile fracture energy of ECC was presented in Figure 2c. From Table 3 and Figure 2c, it can be noticed that the fracture energy increased by 48.5% and 113.7% when the volume content of PVA fiber increased from 1.0% to 1.5% and 2%, respectively. This indicates that increasing the volume content of PVA fiber in the range of 1.0–1.5% can enhance the fracture energy of ECC, which may be because the increase in fiber content increased the bridging action between the fibers and the matrix at the crack, and as a result more energy can be dissipated from cracking to fracture of the ECC. The fracture energy increased by 26.9% and 18.4% when the water-binder ratio increased from 0.24 to 0.25 and 0.28, respectively. This indicates that the energy dissipation capacity of PVA-ECC can be enhanced with an increase in the water-binder ratio, which was unlike PE-ECC (whose energy dissipation capacity increased with increasing water-binder ratio) [41]. Thus, the energy dissipation capacity of PVA-ECC decreased with the decrease of the water-binder ratio.

### 3.2. Stress–Strain Curve and Damage Evolution

**Figure 3:**
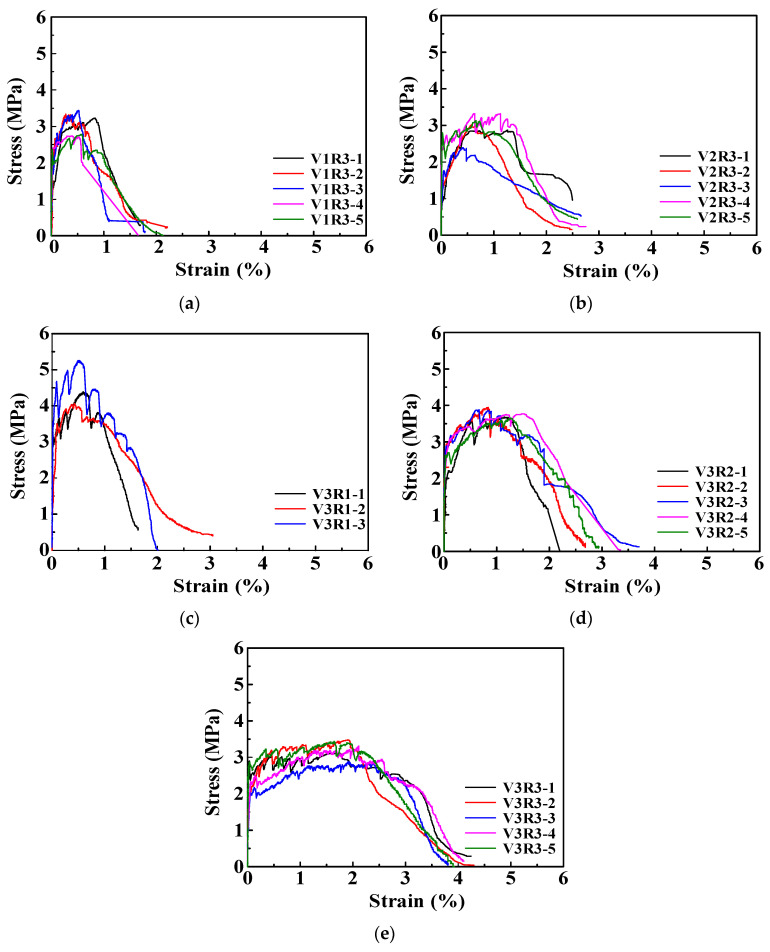
Stress–strain curves of ECC specimens in tension: (**a**) V1R3; (**b**) V2R3; (**c**) V3R1; (**d**) V3R2; (**e**) V3R3.

The uniaxial tensile stress–strain curves of ECC with different fiber volume content and water-binder ratio are shown in Figure 3. As indicated in Figure 3, ECC exhibited obvious strain–hardening characteristic in tension, and after reaching the peak stress, the tensile stress decreased slowly as the strain increased, with a long strain–softening stage, which shows that the ductility of cement mortar matrix can be obviously improved by adding PVA fibers. In addition, the stress–strain curve of the ECC under tension would change significantly when the volume content of fibers or the ECC and the water-binder ratio varied. As shown in Figure 3a,b,e, as the content of PVA fibers increased from 1.0% to 2%, the peak strain, ultimate strain, and the length of the strain–hardening segment increased significantly, but the change of the peak stress is not obvious, which indicates that the PVA fiber mainly plays a role in improving the ductility. As shown in Figure 3c–e, when the water-binder ratio is increased from 0.24 to 0.28, the first cracking stress and the peak stress decrease continuously, while the peak strain and ultimate strain increase significantly, and the length of the strain-hardened section has also increased. This is because the decrease of water-binder ratio would lead to the increase of bond strength between matrix and fibers, which make the fibers vulnerable to rupture rather than being pulled out in tension, and as a result the ductility of specimens reduced [1,2,3].

**Figure 4:**
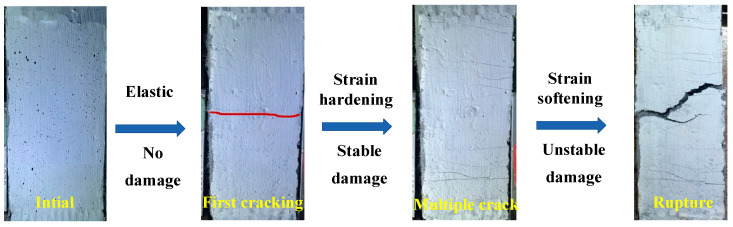
The crack pattern and damage evolution process of the specimens under uniaxial tension.

The crack pattern and damage evolution process of the specimens under uniaxial tension are shown in Figure 4. Based on the damage mechanism and the characteristics of the stress–strain relationship under tension, the uniaxial tensile damage evolution of the ECC can be described in following three stages, including the first stage named elastic nondestructive stage, the second stage named strain-hardening and stable damage stage, and the third stage named strain-softening and unstable damage stage.

The first stage (elastic nondestructive stage) is from the start of loading to the first-cracking point, where the deformation is elastic and the applied external load is mainly borne by the cement mortar matrix. The tensile stress and strain of ECC conform to a linear relationship at this stage and the specimen is basically in a nondestructive state.

The second stage (strain-hardening and stable damage stage) is from the first-cracking point to the peak point. At this stage the tensile stress of ECC increased slowly with increasing strain and the number of cracks increased, showing clear strain-hardening and multiple cracking characteristics. The damage of ECC began to appear at the first-cracking section, but the damage development at the cracked section was restrained to a certain extent due to the bridging effect of PVA fibers. With the increase of stress, new cracks continuously appeared on different sections of ECC, which reflected that the damage was dispersed to multiple cracked sections. As a result, the rate of damage development could be slowed down. While reaching the peak stress, no new cracks were generated, and the restraint effect of fibers on the damage was maximized and the damage development rate was minimized.

The third stage (strain-softening and unstable damage stage) is the stress decreasing segment after the peak point. At this stage, as the tensile strain increased, the tensile stress began to decrease, and the fibers ruptured at cracked sections or were continuously pulled out from the matrix. Besides, the width of cracks gradually increased with the increasing strain. Due to losing the bridging effect of the fibers in the matrix, the damage increased rapidly at this stage, making the damage in the increasing state of nonconvergence. Finally, the main crack of most specimens formed close to the clamp because of the stress concentration close to the clamp, and the ECC specimen ruptured at the main crack.

## 4. Damage Constitutive Model of ECC in Uniaxial Tension

### 4.1. The Proposed Damage Constitutive Model of ECC in Uniaxial Tension

**Figure 5:**
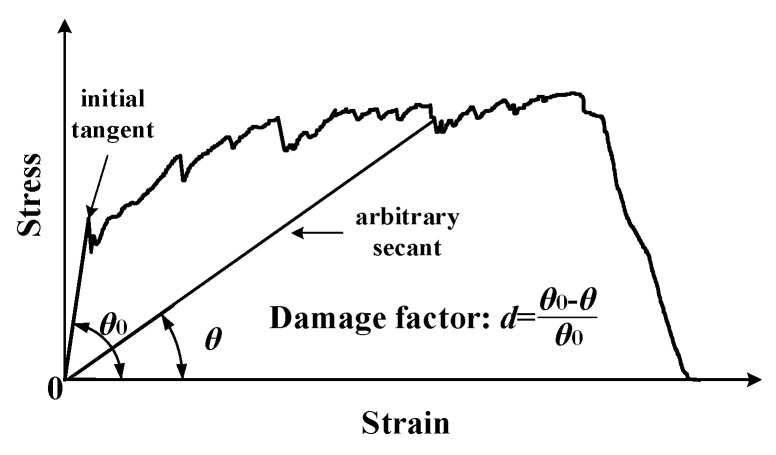
The definition of damage factor of ECC in this paper.

In order to reflect the damage development characteristics of ECC in uniaxial tension at different stages, this paper adopts damage factor (*d*) expressed by the variation of the angle between the secant line of a point of the ECC tensile stress–strain curve and the horizontal axis to represent the degree of damage, as shown in Figure 5.

Therefore, the damage factor can be expressed as:

(5) $$d=\frac{\theta_{0}-\theta }{\theta_{0}}=1-\frac{\theta }{\theta_{0}}$$

where *θ* is the angle between the secant line of a point of the ECC tensile stress–strain curve and the horizontal axis for, *θ*_0_ is the angle between the tensile stress–strain curve of ECC in the elastic nondestructive stage and the horizontal axis. The expressions of *θ*_0_ and *θ* can be given by:

(6) $$\theta_{0}=\arctan\left(E_{0}\right)=\arctan\left(\sigma_{k}/\varepsilon_{k}\right)$$

(7) θ=arctan(E)=arctan(σ/ε)

where *E*_0_ is the initial elastic modulus of ECC, and *E* is the secant modulus of ECC, *σ* and *ε* are the tensile stress and strain of ECC under uniaxial tension, respectively. Thus, the stress–strain relationship of ECC under uniaxial tension can be expressed as

(8) $$\sigma =\tan\left[\left(1-d\right)\arctan\left(E_{0}\right)\right]\varepsilon$$

Based on the analysis of the evolution process of ECC tensile damage, the damage evolution equation is assumed to be constant zero in the elastic section, a power function in the strain–hardening section, and a quadratic polynomial in the strain–softening section, as follows:

(9) $$d=\left\{ \begin{array}{ll} 0 & \left(0\leq \varepsilon \leq \varepsilon_{k}\right)\\ k{\left(\varepsilon -\varepsilon_{k}\right)}^{b} & \left(\varepsilon_{k}<\varepsilon \leq \varepsilon_{p}\right)\\ A\varepsilon^{2}+B\varepsilon +C & \left(\varepsilon_{p}<\varepsilon \leq \varepsilon_{u}\right) \end{array}\right.$$

where *k*, *b*, *A*, *B,* and *C* are model coefficients. In fact, any function capable of describing the damage evolution process of ECC under uniaxial tension can also be used.

Besides, the damage factor (*d*) should satisfy the following boundary conditions:

(10) $$d|{}_{\varepsilon =\varepsilon_{k}}=0$$

(11) $$d|{}_{\varepsilon =\varepsilon_{p}}=k{\left(\varepsilon_{p}-\varepsilon_{k}\right)}^{b}=A\varepsilon_{p}{}^{2}+B\varepsilon_{p}+C\;$$

(12) $$d|{}_{\varepsilon =\varepsilon_{u}}=1=A\varepsilon_{u}{}^{2}+B\varepsilon_{u}+C\;$$

According to Equations (10)–(12), the model coefficients *k*, *B,* and *C* can be obtained by:

(13) $$k=\frac{\arctan\left(\sigma_{k}/\varepsilon_{k}\right)-\arctan\left(\sigma_{p}/\varepsilon_{p}\right)}{{\left(\varepsilon_{p}-\varepsilon_{k}\right)}^{b}\arctan\left(\sigma_{k}/\varepsilon_{k}\right)}$$

(14) $$B=\frac{\arctan\left(\sigma_{p}/\varepsilon_{p}\right)}{\left(\varepsilon_{u}-\varepsilon_{p}\right)\arctan\left(\sigma_{k}/\varepsilon_{k}\right)}-A\left(\varepsilon_{u}+\varepsilon_{p}\right)$$

(15) $$C=1+A\varepsilon_{u}\varepsilon_{p}-\frac{\varepsilon_{u}\arctan\left(\sigma_{p}/\varepsilon_{p}\right)}{\left(\varepsilon_{u}-\varepsilon_{p}\right)\arctan\left(\sigma_{k}/\varepsilon_{k}\right)}$$

### 4.2. Determination of Damage Model Coefficients b and A

From the above expressions for the model coefficients (13)–(15), only two coefficients (*b* and *A*) are independent unknown coefficients. These two coefficients can be determined by regression analysis of test results. Specifically, Equation (9) was used to fit the test result of damage factor–strain curve of each specimen to obtain the values of model coefficients *B* and *A* of each specimen. Then, *b* and *A* are substituted into Equations (13)–(15) to obtain the values of coefficients *k*, *B*, and *C*. The obtained values of model coefficients are given in Table 4.

As can be seen from Table 4, the coefficients *b* and *A* decrease with increasing PVA fiber volume content or water-binder ratio, indicating that both PVA fiber volume content and water-binder ratio influence damage evolution during the strain-hardening and strain-softening stages. Thus, according to regression of test results, the coefficients *b* and *A* can be expressed as a linear function of the PVA fiber volume content (*v*) and the water-binder ratio (*r*) with correlation coefficients R^2^ of 0.989 and 0.994, respectively, which are given as follows:

(16) b=(1.206−0.213v)(2.061r+0.436)

(17) A=(0.358−0.147v)(2.842r+0.247)

where the applicable range of PVA fiber volume content (*v*) is 1–2%, and the applicable range of ECC water-binder ratio (*r*) is 0.24–0.28.

In order to further analyze the characteristic of damage evolution in the strain-hardening and strain-softening stages, the first and second order derivatives of the damage factor expression Equation (9) with respect to strain for these stages are derived as follows:

(18) $$\frac{\partial d}{\partial \varepsilon }=\left\{ \begin{array}{ll} kb{\left(\varepsilon -\varepsilon_{k}\right)}^{b-1} & \left(\varepsilon_{k}<\varepsilon \leq \varepsilon_{p}\right)\\ 2A\varepsilon +B & \left(\varepsilon_{p}<\varepsilon \leq \varepsilon_{u}\right) \end{array}\right.$$

(19) $$\frac{\partial^{2}d}{\partial \varepsilon^{2}}=\left\{ \begin{array}{ll} kb\left(b-1\right)\left(\varepsilon -\varepsilon_{k}\right) & \left(\varepsilon_{k}<\varepsilon \leq \varepsilon_{p}\right)\\ 2A & \left(\varepsilon_{p}<\varepsilon \leq \varepsilon_{u}\right) \end{array}\right.$$

It can be seen from Equations (16)–(19) that in the strain-hardening stage; the first-order derivative of the damage factor is greater than 0, but the second order derivative is less than 0. This indicates that in the strain-hardening stage, as the tensile strain increased, the damage increased continuously, but the increasing rate of damage decreased due to the presence of fibers, and the damage in a stable development state. In the strain-softening stage, the first and second order derivatives of the damage are both greater than 0. This demonstrates that in the strain-softening stage, as the tensile strain increased, the damage of the ECC increased continuously, and the increasing rate also increased due to the continuous pulling out or rupture of the PVA fibers, and the damage is in an unstable development state. The above analysis is consistent with the test results of the evolutionary mechanism of damage in Section 3.2, which also indicates that the damage factor expressed by Equation (9) proposed in this paper can reasonably characterize the evolutionary law of the damage of ECC in uniaxial tension.

### 4.3. Model Validation

In order to verify the applicability of the proposed model, existing experimental results in reference [43] will be validated. The average test values [43] of performance indicators of each group were presented in Table 5. In order to validate the proposed calculation formula for damage model coefficients, *b*, and *A*, the measured values (*b_expt_* and *A_expt_*) of the validation group were compared with the calculated values by using Equations (16) and (17) (*b_calc_* and *A_calc_*), as shown in Table 5. As can be seen from Table 5, the mean values of the ratio of measured value to calculated value for damage model coefficients, *b*, and *A*, are 0.971 and 1.155 respectively, with coefficients of variation of 0.023 and 0.208, which indicates that the Equations (16) and (17) are acceptable for calculating the damage model coefficients (*b* and *A*).

**Figure 6:**
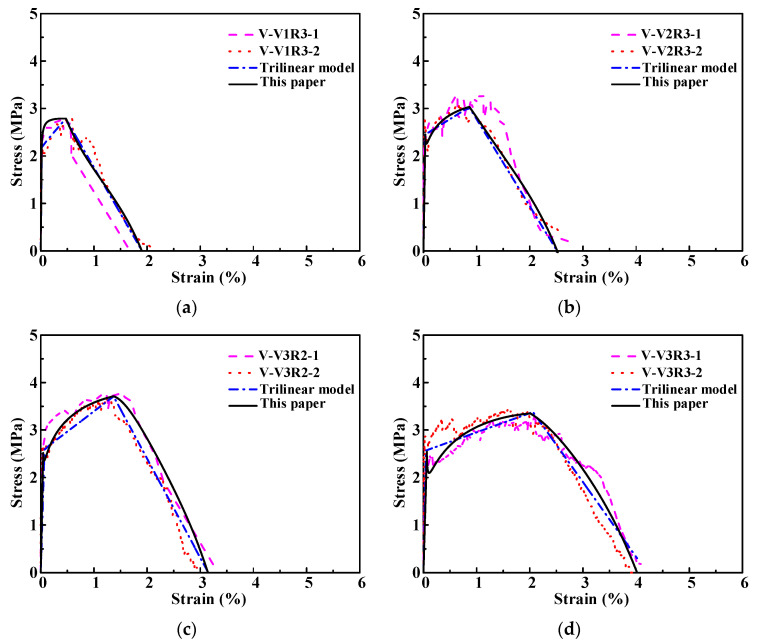
Tensile stress strain curves of: (**a**) Group V-V1R3; (**b**) Group V-V2R3; (**c**) Group V-V3R2; (**d**) Group V-V3R3.

In order to verify the accuracy of the damage constitutive model of ECC in uniaxial tension proposed in this paper, the predicted stress–strain curves for ECC under uniaxial tension were obtained by using the proposed model [Equations (9) and (10)] and expressions of model coefficients [Equations (13)–(17)], and were compared with test stress–strain curves and the existing trilinear model [23,24,25], as shown in Figure 6. The expression of the trilinear model [23,24,25] is given as follows:

(20) $$\sigma =\{\begin{array}{ll} \frac{\sigma_{k}}{\varepsilon_{k}}\varepsilon & \left(\varepsilon \leq \varepsilon_{k}\right)\\ \sigma_{k}+\left(\frac{\sigma_{p}-\sigma_{k}}{\varepsilon_{p}-\varepsilon_{k}}\right)\left(\varepsilon -\varepsilon_{k}\right) & \left(\varepsilon_{k}<\varepsilon \leq \varepsilon_{p}\right)\\ \sigma_{p}-\frac{\sigma_{p}}{\varepsilon_{u}-\varepsilon_{p}}\left(\varepsilon_{u}-\varepsilon \right) & \left(\varepsilon_{p}<\varepsilon \leq \varepsilon_{u}\right) \end{array}$$

As can be seen from Figure 6, in the elastic nondestructive stage, both the proposed model in this paper and the trilinear model are in good agreement with the test curves. In the strain-hardening stage, the model proposed in this paper can describe accurately the characteristic of the test stress–strain curves, which rise nonlinearly with the reducing slope, whereas the trilinear model [23,24,25] cannot give accurate prediction of test curves in this stage. In the strain-softening stage (unstable damage), the model proposed this paper can reflect the obvious nonlinear characteristic of test stress–strain curves with fist relatively slow decent rate and then fast decent rate, which cannot be characterized by the trilinear model although the discrepancy between the trilinear model and test curves are relatively small.

## 5. Application of the Proposed Model to Finite Element Modelling

### 5.1. Establishment of Nonlinear Finite Element Model

**Figure 7:**
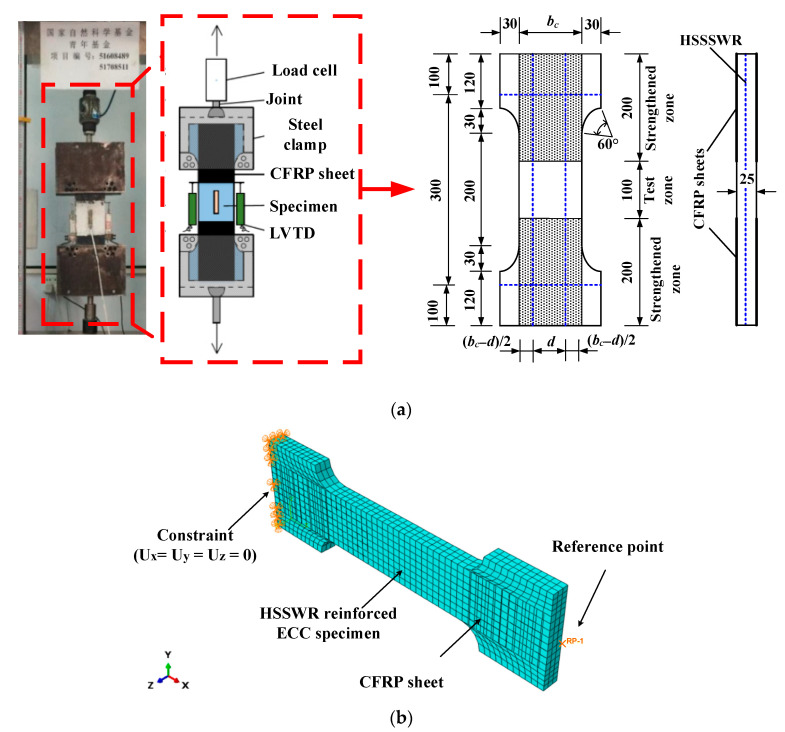
Test setup and 3D FE model of specimens: (**a**) test setup and specimen details (The Chinese project fund in the picture is not related to the research in this paper); (**b**) 3D FE model.

In order to further prove the applicability of the proposed damage constitutive model of ECC in uniaxial tension, this model was incorporated to the FE analysis of the tensile performance of high-strength stainless steel wire ropes (HSSWR) reinforced ECC by using the general FE analysis software ABAQUS. The FE simulation results of by adopting this model would be compared with the results of the uniaxial tension tests on HSSWR reinforced ECC performed by the authors’ research group [44] and the FE simulation results by using the trilinear model of ECC [23,24,25]. The test setup and specimen details are shown in Figure 7 and Table 6, which were detailed in the literature [44]. The volume content of PVA fibers (*v*), the water-binder ratio (*r*), first-cracking strain (*ε_k_*), first-cracking stress (*σ_k_*), peak strain (*ε_p_*), peak stress (*σ_p_*), and ultimate strain (*ε_u_*) of the used ECC are 2%, 0.28, 0.02%, 2.45 MPa, 2.79%, 3.53 MPa and 4%, respectively. The maximum tensile stress (*σ_st_*) and its corresponding strain (*ε_st_*) of the used HSSWR with a nominal diameter of 2.4 mm are 1573 MPa and 2.99%, respectively. The HSSWR reinforced ECC specimen is of the shape of dumbbell, and the HSSWR are symmetrically arranged at the middle height of ECC plate section with equal spacing, as shown in Figure 7a. In order to prevent the end of the specimen from local crushing by the clamp during the loading process, two layers of CFRP sheets were bonded on the surfaces of two ends of the specimen, as shown in Figure 7a.

The three-dimension (3D) nonlinear FE model was used to simulate the tension tests on the HSSWR reinforced ECC specimens, as shown in Figure 7b. In this model, the ECC, HSSSWR and CFRP sheets were assumed to be homogeneous materials and modelled by using eight-node solid elements (C3D8R), two-node truss element (T3D2), and plane shell element, which are available in the FE package of ABAQUS [45].

The mesh size of the elements of ECC and HSSSWR was set to be 10 mm. The interface between ECC and CFRP sheets was assumed to have no relative slip and connected by using the tie constraint feature of ABAQUS [45]. The embedded region constraint feature of ABAQUS [45] was used to prescribe the collaborative deformation between ECC and HSSSWR.

Both the proposed damage constitutive model [Equations (8), (9), and (13)–(17)] and trilinear model [23,24,25] were adopted to characterize the stress–strain relationship of ECC based on the ABAQUS concrete damage plastic model [45]. Besides, the material parameters dilation angle, flow potential eccentricity, biaxial to uniaxial compression plastic strain ratio, invariant stress ratio, and viscosity were set to, 35°, 0.1, 1.07, 0.6667, and 0.0005, respectively.

The tensile constitutive model of HSSSWR proposed by the authors’ previous studies [44] was adopted to define the stress–strain relationship of HSSSWR in tension based on the ABAQUS Von-Mises plastic model [45], which is expressed as follows:

(21) $$y=3.33x+3.66x^{2}+1.33x^{3}$$

where *x* = *ε_s_*/*ε_st_*, *y* = *σ_s_*/*σ_st_*, and *σ_s_* and *ε_s_* indicate the tensile stress and strain of HSSSWR, respectively.

The constraint conditions of the specimen were reproduced by constraining the three translational freedom degrees of one end of the specimen and applying loading by using displacement control at the reference point (RP) created at the section centroid of the other end ECC surface at the loaded end were. The details of 3D nonlinear FE modeling of HSSWR reinforced ECC specimens are shown in Table 6 and Figure 7b.

### 5.2. Comparison of Simulated Results and Test Results

**Figure 8:**
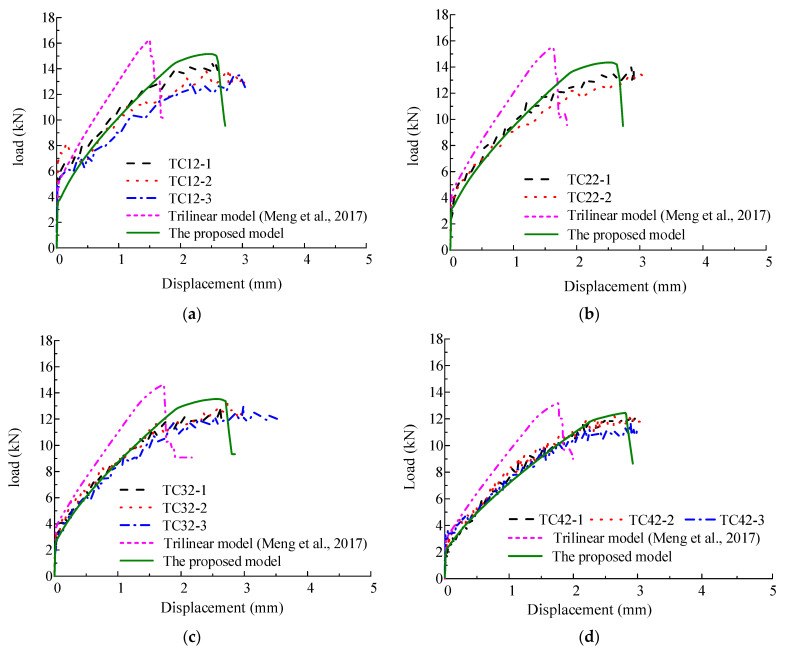
Comparison of tensile load-displacement curves of HSSWR reinforced ECC: (**a**) TC-1 group; (**b**) TC-2 group; (**c**) TC-3 group; (**d**) TC-4 group [23,24,25].

The predicted load-displacement curves of HSSWR reinforced ECC specimens in tension through FE method by using the proposed model in this paper and the trilinear model [23,24,25] are compared with test results, as shown in Figure 8. It can be seen in Figure 8 that the predicted results by FE method using the proposed model are in good agreement with test results. Whereas, although before ECC cracking (when the curves are almost linear), the FE simulation results by using the trilinear model [23,24,25] agree well with test results, after cracking of ECC (when the curves enter the nonlinear phase), the FE simulation results by using the trilinear model [23,24,25] cannot reflect the nonlinear characteristic of the curves. Besides, the predicted maximum loads by using the trilinear model [23,24,25] are obviously larger than the test result, while the predicted strains corresponding to the maximum loads are significantly smaller than the test results. The ratio of the predicted maximum loads by using the proposed model and the trilinear model [23,24,25] to the test result are 1.042 and 1.123, with variation coefficient of 0.026, respectively. The ratio of the predicted displacement corresponding to the maximum load by using the proposed model and the trilinear model [23,24,25] to the test result are 0.964 and 0.574, with variation coefficient of 0.028 and 0.076, respectively. This demonstrates that the proposed damage constitutive model of ECC in uniaxial tension in this paper can give more accurate prediction of tensile behavior of ECC and can be accepted for characterizing the damage evolution of ECC.

## 6. Conclusions

Through the uniaxial tensile test study of ECC specimens with test variables of PVA fiber volume content and water-binder ratio, the effects of the above variables on the uniaxial tensile performance of ECC and the whole process damage mechanism of ECC were revealed, based on which a damage constitutive model of ECC in uniaxial tension was developed. Finally, by applying it to the finite analysis of tensile properties of the new composite material HSSWR-ECC, the acceptability of this model was verified. The following conclusions can be drawn:

(1) An increase in the volume content of PVA fibers could enhance the cracking strain, cracking stress, peak strain, ultimate strain, and fracture energy of ECC. An increase in the water-binder ratio could reduce the cracking stress and peak stress, but would increase the cracking strain, peak strain, ultimate strain, deformation capacity, and fracture energy;
(2) The damage development process of ECC under uniaxial tension can be divided into three stages: (1) elastic undamaged stage, (2) strain hardening and stable damage stage, and (3) strain-softening and nonconverging damage stage. Adding PVA fibers in ECC could disperse the damage in ECC, and could obviously reduce the damage development rate in the strain hardening and stable damage stage. While reaching the peak stress, the damage development rate was minimized;
(3) By comparing with the experimental results, the damage constitutive model and expressions of model parameters for ECC in uniaxial tension proposed in this paper, which considered the effects of PVA fiber volume content and water-binder ratios, was verified to be able to give accurate prediction of the nonlinear stress–strain relationship of ECC, reflecting the damage evolution characteristic of ECC at different stressing stages;
(4) Based on FE analysis, the model in this paper can be applied to simulate the nonlinear tensile process of HSSWM-ECC. Compared with the trilinear model used by ECC, the model in this paper has higher accuracy and smaller discreteness;
(5) Due to the limitations of the experimental research in this paper, the range of experimental parameters and the number of specimens should be expanded in the future to make the proposed model parameters more accurate. Secondly, the experiments under repeated loads should be explored, and the damage constitutive model of ECC under repeated loads should be proposed. Finally, it may be more meaningful and challenging to apply the proposed model to the analysis of real structures.

## Figures and Tables

**Table 1:** ECC mix proportion.

Group Number	Cement	Silica Sand	Fly Ash	Micro-Silica Fume	Water	PVA Fiber	Superplasticizer
V1R3	1.000	1.500	2.000	0.073	0.860	0.029	0.041
V2R3	1.000	1.500	2.000	0.073	0.860	0.044	0.041
V3R1	1.000	1.500	2.000	0.073	0.738	0.057	0.041
V3R2	1.000	1.500	2.000	0.073	0.768	0.057	0.041
V3R3	1.000	1.500	2.000	0.073	0.860	0.057	0.041

**Table 2:** Performance indicators of PVA.

Fiber	Length (mm)	Diameter (μm)	Tensile Strength (MPa)	Elastic Modulus (GPa)	Elongation (%)	Density (g/cm^3^)
PVA	12	40	1560	41	6.5	1.3

**Table 3:** Details of specimens and test results.

Group	Specimen ID	*v* (%)	*r*	*ε_k_*/ (%)	*σ_k_*/ MPa	*ε_p_*/ (%)	*σ_p_*/ MPa	*ε_u_*/ (%)	*G_f,N_* (kJ/m^2^)
V1R3	V1R3-1	1	0.28	0.037	1.347	0.814	3.226	1.697	3.044
	V1R3-2			0.039	2.407	0.274	3.338	2.211	3.950
	V1R3-3			0.015	1.797	0.518	3.438	1.786	3.681
	V1R3-4			0.019	1.960	0.385	2.740	1.652	2.976
	V1R3-5			0.018	2.370	0.563	2.780	2.128	3.913
Average value				0.025	1.976	0.511	3.104	1.895	3.513
Standard deviation				0.011	0.439	0.204	0.324	0.257	0.471
V2R3	V2R3-1	1.5	0.28	0.072	1.746	1.254	2.867	2.498	5.705
	V2R3-2			0.050	1.259	0.575	3.012	2.485	4.841
	V2R3-3			0.055	2.128	0.420	2.792	2.663	5.458
	V2R3-4			0.040	2.166	1.068	2.997	2.461	4.761
	V2R3-5			0.025	2.799	0.663	3.130	2.590	5.313
Average value				0.048	2.020	0.796	2.959	2.539	5.216
Standard deviation				0.017	0.569	0.351	0.132	0.084	0.405
V3R1	V3R1-1	2	0.24	0.033	3.274	0.590	4.392	1.633	7.181
	V3R1-2			0.043	3.508	0.425	4.067	3.050	6.808
	V3R1-3			0.048	3.538	0.495	5.256	2.008	8.270
Average value				0.041	3.440	0.503	4.572	2.230	7.420
Standard deviation				0.008	0.144	0.083	0.613	0.734	0.757
V3R2	V3R2-1	2	0.25	0.090	2.256	1.140	3.680	2.200	6.656
	V3R2-2			0.066	2.960	0.811	3.949	2.682	9.934
	V3R2-3			0.088	3.074	0.665	3.884	3.705	11.000
	V3R2-4			0.040	2.462	1.476	3.769	3.350	10.140
	V3R2-5			0.053	2.574	1.265	3.655	2.940	9.344
Average value				0.067	2.665	1.071	3.787	2.975	9.415
Standard deviation				0.022	0.343	0.331	0.127	0.583	1.653
V3R3	V3R3-1	2	0.28	0.033	2.900	1.755	3.111	4.245	10.166
	V3R3-2			0.092	2.216	1.908	3.477	4.298	12.673
	V3R3-3			0.092	2.033	2.317	2.883	3.808	10.053
	V3R3-4			0.075	2.256	2.110	3.309	4.108	11.764
	V3R3-5			0.055	2.827	1.910	3.390	3.929	11.065
Average value				0.069	2.447	2.000	3.234	4.077	11.144
Standard deviation				0.026	0.391	0.217	0.238	0.208	1.104

**Table 4:** Damage model coefficients.

Group	V1R3	V2R3	V3R1	V3R2	V3R3
*v* (%)	1	1.5	2	2	2
*r*	0.28	0.28	0.24	0.25	0.28
*b*	0.997	0.877	0.915	0.844	0.784
*A*	0.214	0.132	0.197	0.151	0.067
*k*	0.198	0.199	0.127	0.162	0.203
*B*	0.138	0.045	0.004	−0.090	−0.090
*C*	−0.030	0.035	0.011	0.108	0.252

**Table 5:** Test results and damage model coefficients of the verification groups.

Group	*v* (%)	*r*	*ε_k_* (%)	*σ_k_* (MPa)	*ε_p_* (%)	*σ_p_* (MPa)	*ε_u_* (%)	*b_expt_*	*b_calc_*	*A_expt_*	*A_calc_*
V-V1R3	1	0.28	0.018	2.165	0.474	2.760	1.890	0.993	0.997	0.216	0.214
V-V2R3	1.5	0.28	0.033	2.483	0.864	3.063	2.526	0.915	0.877	0.141	0.132
V-V3R2	2	0.25	0.047	2.518	1.371	3.712	3.145	0.880	0.844	0.122	0.151
V-V3R3	2	0.28	0.065	2.542	2.010	3.349	4.018	0.816	0.784	0.046	0.067

**Table 6:** HSSWR-ECC Specimen parameter details.

Specimen ID	*v* (%)	*r*	Test Section Width, *b_c_* (mm)	HSSWR Spacing, *d* (mm)	HSSWR Diameter (mm)	HSSWR Reinforcement Ratio (*%*)
TC1	2	0.28	80	50	2.4	0.28
TC2	2	0.28	70	40	2.4	0.32
TC3	2	0.28	60	30	2.4	0.37
TC4	2	0.28	47	20	2.4	0.48

## Data Availability

All the data is available within the manuscript.
